# Evaluation of the Bioaccessibility of Antioxidant Bioactive Compounds and Minerals of Four Genotypes of *Brassicaceae* Microgreens

**DOI:** 10.3390/foods8070250

**Published:** 2019-07-09

**Authors:** Beatriz de la Fuente, Gabriel López-García, Vicent Máñez, Amparo Alegría, Reyes Barberá, Antonio Cilla

**Affiliations:** 1 Nutrition and Food Science Area, Faculty of Pharmacy, University of Valencia, Av. Vicent Andrés Estellés s/n, Burjassot, 46100 Valencia, Spain; 2 CIAM (Centro de Innovación Agronómico_Grupo Alimentario Citrus), Avda. dels Gremis, Parcela 28. Pol. Ind. Sector 13, Riba-roja de Túria, 46394 Valencia, Spain

**Keywords:** microgreens, *Brassicaceae*, bioaccessibility, minerals, bioactive compounds, antioxidants

## Abstract

Microgreens constitute an emerging class of fresh, healthy foods due to their nutritional composition. In this study the content of minerals and antioxidant bioactive compounds, and for the first time bioaccessibility, were evaluated in broccoli (*Brassica oleracea* L. var. *italica* Plenck), green curly kale (*Brassica oleracea* var. *sabellica* L.), red mustard (*Brassica juncea* (L.) Czern.) and radish (*Raphanus sativus* L.) hydroponic microgreens. Macro- (K, Ca, Mg) and oligo-elements (Fe, Zn), ascorbic acid, total soluble polyphenols, total carotenoids, total anthocyanins, total isothiocyanates and total antioxidant capacity (Trolox Equivalent Antioxidant Capacity and Oxygen Radical Absorbance Capacity) were determined before and after the standardized simulated gastrointestinal digestion process. All microgreens provided relevant amounts of vitamin C (31–56 mg/100 g fresh weight) and total carotenoids (162–224 mg β-carotene/100 g dry weight). Mineral content was comparable to that normally found in hydroponic microgreens and the low potassium levels observed would allow their dietetic recommendation for patients with impaired kidney function. Both total soluble polyphenols and total isothiocyanates were the greatest contributors to the total antioxidant capacity after digestion (43–70% and 31–63% bioaccessibility, respectively) while macroelements showed an important bioaccessibility (34–90%). In general, radish and mustard presented the highest bioaccessibility of bioactive compounds and minerals. Overall, the four hydroponic *Brassicaceae* microgreens present a wide array of antioxidant bioactive compounds.

## 1. Introduction

Microgreens are a new class of small, fresh, edible vegetables considered as a good nutritional source because of their high mineral and bioactive compound content. The meaning of microgreen refers to immature greens harvested at soil level between the first and third week after sowing, when the cotyledon is fully developed and the first true leaves have emerged [1,2,3], being different from both baby leaf (cut greens for salads) [4] and sprouts (germinated seeds with entire roots) [5]. Microgreens can be produced from many vegetables, herbaceous plants, aromatic herbs, grains and wild species [6,7,8], and possess distinctive organoleptic properties, such as color, shape, texture and taste [2,8,9].

These new and young vegetables are a versatile, nutritive and sustainable crop from cultivation to consumption. They can be adapted to different agronomic practices to obtain a final product which is of high organoleptic and nutritional quality [1]. Likewise, growing conditions (soil, compost, hydroponic) directly affect the plant growth and the levels of phytonutrients and minerals [5,10]. In this sense, soilless or hydroponic is based on the use of nutrient solution rather than soil for crop production, reducing fertilizer and water resources as well as the use of pesticides [11]. According to Weber [10], a higher amount of minerals was obtained in broccoli microgreens compared to the mature vegetable using about 200 times less water, 94% less time and without applying fertilizer, pesticides or energy-demanding transport. Besides the possibility of saving natural resources and chemicals, the production and consumption of microgreens have additional advantages, turning these products into a new, healthy, and environmentally-friendly vegetable option. For instance, the containerized production in an industrial, local or home scale implies that the final consumer can harvest them just at the moment of being used, and their consumption only without roots generates much less waste than adult vegetables [1,10,12].

In addition, microgreens have been considered as healthy foods because of their general higher levels of phytochemicals with respect to their mature counterparts [2,6,7,12]. In this context, a recent review has defined microgreens as a new food for the 21st century attributing them a potential role as anti-inflammatory, anti-carcinogenic, anti-obesogenic and anti-atherosclerotic [5]. In contrast to the great amount of nutrients expected to obtain health benefits, Renna et al. [13] developed chicory and lettuce microgreens with a reduced potassium content to be consumed by chronic kidney disease patients. Also, microgreens have been proposed as ideal food for people with a vegetable-based diet such as vegans or vegetarians, and even for space crew members due to their limited access to food diversity [14].

It is known that *Brassica* vegetables, at the mature stage, contain beneficial nutrients for human health [15], and available data reveal that their intake reduces the risk of chronic diseases [16]. Probably this is the reason why among the different species used to obtain microgreens, the *Brassicaceae* family is one of the most widely grown to date [2]. Nevertheless, information in the literature about *Brassicaceae* microgreens is limited regarding the concentrations of the antioxidant bioactive compounds and minerals that were examined in this work. There are some studies on this subject in broccoli [7,10,17,18,19,20], kale [19,20,21], mustard [6,8,9,20,22,23,24] and radish [6,8,20,24,25,26] microgreens. However, the health-related effects of bioactive compounds of a food depend not only on their content and the amount consumed, but also on their bioavailability. Although in vivo assays are the gold standard for this purpose, these studies are expensive, lengthy, and have some ethical concerns. In turn, in vitro digestion allows one to estimate the bioaccessibility (the total amount of a food compound in soluble form and released from the solid food matrix that is available for absorption) [27], a prerequisite of bioavailability.

The aim of the present study was to evaluate the content and for the first time the bioaccessibility of the main antioxidant bioactive compounds (ascorbic acid, total carotenoids, total isothiocyanates, total anthocyanins, total soluble polyphenols), total antioxidant capacity, as well as macro- (K, Ca, Mg) and oligoelements (Fe, Zn) provided by the four studied hydroponic *Brassicaceae* microgreens: broccoli, kale, mustard and radish.

## 2. Materials and Methods

### 2.1. Plant Material and Sample Preparation

Four microgreen species belonging to the *Brassicaceae* family were evaluated in this study: broccoli (*Brassica oleracea* L. var. *italica* Plenck), green curly kale (*Brassica oleracea* var. *sabellica* L.), red mustard (*Brassica juncea* (L.) Czern.) and radish (*Raphanus sativus* L.). Mustard and radish cultivar seeds were purchased from CN Seeds Ltd. (Cambridgeshire, UK) and kale and broccoli from Rocalba S.A. (Huesca, Spain) and Intersemillas S.A. (Valencia, Spain), respectively.

Microgreens were produced by the Agronomic Innovation Center (CIAM) of Grupo Alimentario Citrus Company (Valencia, Spain) at the end of August 2017. A hydroponic system was created by placing substrates of pine tree fibers (12 cm × 12 cm × 0.4 cm) on plastic trays. Two seeding densities were selected: 3.8 seeds cm^−2^ for broccoli and kale and 2.8 seeds cm^−2^ for mustard and radish. The sown substrates were moistened with water and introduced into a growth chamber at 18 °C and 90% relative humidity (RH) until the germination of the seeds. Then, they were moved into an unheated greenhouse where no artificial light treatment was applied. The incidence of natural light at this time of year provided a daily average of 18 °C and 61% RH. The following nutritive solution expressed as mmol/L for each component was applied daily: NO_3_^−^ (5.3), H_2_PO_4_^−2^ (1.5), SO_4_^−2^ (4.4), HCO_3_^−^ (0.5), Cl^−^ (5.3), K^+^ (1.5), Ca^+2^ (6.3), Mg^+2^ (1.3) and Na^+^ (3.1). An average fertigation value of 20.4 l m^−2^ per day from June to September 2017 was recorded. No phytosanitary treatment was used.

Nine days after seeding for radish and 7 days for broccoli, kale and mustard, the microgreens were transported in plastic trays (58 cm × 39 cm) from CIAM to the University of Valencia (UV). They were fertigated just before being moved in order to maintain good humidity conditions during the 30 min period of transportation. In our laboratory at the UV, a total of 40 trays were received (8 for kale, 12 for mustard and 10 for broccoli and radish). For each microgreen, approximately 400 g were harvested as close as possible to the root using sterilized scissors. Next, a pool was made to homogenize each microgreen sample, and then they were randomly divided into several replicates. Fresh microgreens were used immediately for ascorbic acid analysis and the rest of the collected samples were weighted inside aluminum containers before freezing at −80 °C. Frozen microgreens were lyophilized for 48 h (Sentry 2.0 Virtis SP Scientific, Philadelphia, PA, USA) and maintained in a desiccator until constant weight to obtain dry weight (DW) percentage (4.76 ± 1.43, 4.71 ± 1.49, 4.25 ± 1.36 and 4.91 ± 1.55 for broccoli, kale, mustard and radish, respectively) in accordance with the range 3.9–8.1% described in previous studies on these *Brassicaceae* microgreens [6,7,8,19]. Next, samples were ground into a fine powder in a grinder (Super Junior “S” Moulinex, Alençon, France) and stored at −20 °C for subsequent experiments.

### 2.2. Reagents

#### 2.2.1. In Vitro Gastrointestinal Digestion

Pepsin (porcine, 975 units per mg protein), pancreatin (porcine, activity equivalent to 8 × USP specifications), bile extract (porcine), ammonium carbonate ((NH_4_)_2_CO_3_), sodium bicarbonate (NaHCO_3_) and calcium chloride dihydrate (CaCl_2_(H_2_O)_2_) were purchased from Sigma Chemical Co. (St. Louis, MO, USA). Potassium chloride (KCl), sodium chloride (NaCl), magnesium chloride hexahydrate (MgCl_2_(H_2_O)_6_) and potassium dihydrogen phosphate (KH_2_PO_4_) were supplied by Merck (Darmstadt, Germany). Culture-grade water was obtained from B. Braun (Melsungen AG, Germany). Simulated Salivary Fluid (SSF), Simulated Gastric Fluid (SGF), Simulated Intestinal Fluid (SIF) and enzymatic activity assays were prepared according to Minekus et al. (2014). A water bath with orbital shaking (Stuart SBS30, Staffordshire, UK) and centrifuge (Eppendorf 5810, Hamburg, Germany) were used to simulate the gastrointestinal digestion process.

#### 2.2.2. Bioactive Compounds and Antioxidant Capacity

Glacial acetic acid, metaphosphoric acid, formic acid and L (+) ascorbic acid (≥99%) were supplied by Panreac Química (Barcelona, Spain). Sodium salt dihydrate 2,6-dichlorophenolindophenol (DCFI), Tris(hydroxymethyl)aminomethane, potassium phosphate monobasic (Na_2_HPO_4_), potassium phosphate dibasic (K_2_HPO_4_), sodium phosphate dibasic (Na_2_HPO_4_), potassium chloride (KCl) and sodium acetate (C_2_H_3_NaO_2_) were obtained from Merck (Darmstadt, Germany). Sodium carbonate (Na_2_CO_3_), potassium persulfate (K_2_S_2_O_8_), Folin-Ciocalteu reagent, 2,2′-azino-bis-(3-ethylbenzothiazoline-6-sulphonic acid) (ABTS), 2,2′-azobis-(2-amidinopropane) dihydrochloride (AAPH), 6-hydroxy-2,5,7,8-tetramethylchroman-2-carboxylic acid (Trolox), gallic acid and sulforaphane standard (≥90%) were purchased by Sigma Chemical Co. (St. Louis, MO, USA). Ethanol (96%), methanol and n-hexane (96%) were provided from Scharlau (Barcelona, Spain). Sodium fluorescein was obtained from Fluka Chemie AG (Bunds, Switzerland) and 1,2-benzenedithiol (BDT) (96%) from Acros organics (BVBA Thermo Scientific, Geel, Belgium). Water was purified by a Milli-Q system (Milford, MA, USA).

#### 2.2.3. Minerals

Titrisol concentrated standards (1000 mg) of macro and oligoelements (Ca, Mg, K, Fe and Zn) and nitric acid (HNO_3_) (65%) were purchased by Merck (Darmstadt, Germany) while hydrochloric acid (37%) was obtained from Scharlau (Barcelona, Spain).

### 2.3. Methodology for In Vitro Gastrointestinal Digestion

Freeze-dried samples were rehydrated to their original moisture contents in order to be as close as possible to the edible fresh microgreen [28], and in vitro gastrointestinal digestion based on the standardized method INFOGEST [29] was applied. Because of the absence of starch in the samples, the salivary step was carried out without α-amylase enzyme. Briefly, 5 g of rehydrated microgreen or culture-grade water (blank of digestion), 3.5 mL of SSF, 25 µL of 0.3 M CaCl_2_ and culture-grade water to a final volume of 10 mL were mixed by mechanical shaking at 95 opm and 37 °C for 2 min. Immediately afterwards, to simulate the gastric phase, 7.5 mL of SGF, 1.6 mL of pepsin solution (25,000 U/mL) and 5 µL of 0.3M CaCl_2_ were added to the gastric solution obtained and agitated for 1 min. The pH was adjusted at 7.0 ± 0.1 with 6M NaOH and culture-grade water was added up to a final volume of 40 mL. The intestinal mixture was incubated again at 95 opm and 37 °C for 2 h and after this period the digested samples were cooled in an ice bath and centrifuged at 3100 *g* and 4 °C for 90 min to obtain the bioaccessible fraction (BF). The values of the blank of digestion obtained in each assay were subtracted from the values of the digested microgreen samples to remove possible interferences caused by digestive enzymes or simulated fluids, in order to avoid overestimation of results. The results of bioaccessibility were calculated as the ratio between the concentration of each bioactive compound in the BF and the initial concentration in microgreens. The results were expressed as percentage of bioaccessibility according the next Equation (1):

Bioaccessibility (%) = (content in BF/initial content) × 100(1)

### 2.4. Analysis of Bioactive Compounds

#### 2.4.1. Ascorbic Acid

Total ascorbic acid (AA) was determined by the AOAC Official Method 967.21 [30] and the procedure applied by Xiao et al. [8] was used to obtain extracts from fresh samples. Just harvested microgreens (6 g) and ice-cold 5% (*w*/*v*) metaphosphoric acid (20 mL) were homogenized in a Polytron (PT 2000 AFORA S.A. Kinematica, Switzerland) at 15,000 rpm for 1 min, centrifuged at 3000 *g* for 20 min and 4 °C, and filtered through Whatman nº 4 filter paper. BF samples were directly used for the titrimetric method. Both kind of samples were mixed 1:1 (*v*/*v*) with acetic acid—Metaphosphoric acid and the amount of acid ascorbic was measured using 2,6-DCFI. Concentration of AA was calculated by using L (+)-ascorbic acid standard solution (1 mg/mL). The results were expressed as mg AA/100 g fresh weight (FW).

#### 2.4.2. Total Carotenoids

Total carotenoids were extracted as described by Sims and Gamon [31]. Quantification for extracts and BF was determined spectrophotometrically according to Sotelo et al. [32]. Dry microgreen powder (10 mg) was ground in 30 mL cold 80/20 (*v*/*v*) acetone/Tris buffer solution (pH 7.4) and mixed overnight in darkness at room temperature. Afterwards, samples were centrifuged at 3100 *g* for 10 min and supernatants were diluted 1/6 (*v*/*v*) in acetone/Tris buffer solution before measuring absorbance at 470, 537, 647 and 663 nm. Carotenoid content was obtained by following the next Equation (2) and the results were expressed as mg of β-carotene/100 g DW.

Carotenoids = (A_470_ − (17.1 × (Chl_a_ + Chl_b_) − 9.479 × Anthocyanin))/119.26 (2)

where,

Anthocyanin = 0.8173 A_663_ − 0.00697 A_647_ − 0.002228 A_663_
Chla = 0.013773 A_663_ − 0.000897 A_537_ − 0.003046 A_647_
Chlb = 0.024054 A_647_ − 0.004305 A_537_ − 0.005507 A_663_

#### 2.4.3. Total Isothiocyanates

The extraction of total isothiocyanates was performed as described by Torres-Contreras et al. [33]. Freeze-dried samples (100 mg) and water (5 mL) were mixed and centrifuged at 12,000 *g* for 8 min. The supernatant was diluted 1/5 (*v*/*v*) in water and 100 µL was used for cyclocondensation reactions [34]. BF samples were directly used for cyclocondensation reactions. Umber tubes were used and the order for the mixture was the following: 900 µL of 100 mM potassium phosphate buffer solution (pH 8.5), 900 µL of methanol, 100 µL of the isothiocyanate extract dilution and 100 µL of 80 mM 1,2-BDT in methanol to initiate the reaction. The tubes were heated at 65 °C for 1 h and cooled at room temperature before measuring absorbance at 365 nm. A standard curve of DL-sulforaphane in the range of 25–800 mg/L was subjected to the same analysis conditions and the results were expressed as mg of sulforaphane/100 g of DW.

#### 2.4.4. Total Anthocyanins

Anthocyanin pigments were extracted according to Hanlon and Barnes [25] with some modifications and total anthocyanin content was determined by the pH differential method [35]. Anthocynins from lyophilized microgreens (400 mg) were extracted with a 0.1% (*w*/*w*) acetic acid aqueous solution (4 mL) in a sonicator for 10 min. After centrifugation at 3100 *g* for 10 min at 4 °C and filtering through a Whatman nº 4 filter paper, the extract of microgreens or BF were diluted 1/5 (*v*/*v*) in two different buffer solutions (0.025 M potassium chloride pH 1 and 0.4 M sodium acetate pH 4.5). Absorbance of diluted samples in both buffers was measured at 520 and 700 nm. The anthocyanin concentration was calculated according to the following Equation (3), and the final results were expressed as mg of cyanidin-3-glucoside/100 g DW.

Anthocyanin pigment = A × MW × DF × 10^3^/ε × l (3)

where

A = (A520 − A700) pH1 − (A520 − A700) pH 4.5
MW (molecular weight for cyanidin-3-glucoside) = 449.2 g/mol
DF (dilution factor) = 5
10^3^ = factor for conversion from g to mg
ε = 26,900 molar extinction coefficient
l = path length in cm

#### 2.4.5. Total Soluble Polyphenols

The total soluble polyphenols content was analyzed by the Folin-Ciocalteu method with some modifications [36], and extraction was carried out according to the method described by Xiao et al. [26]. Briefly, 100 mg of lyophilized microgreen sample was mixed with 10 mL of 80% methanol and sonicated for 30 s. Then, a hexane wash procedure was applied three times (4 mL of hexane was added, sonicated again for 30 s, centrifuged at 6650 *g* for 5 min at 4 °C and the hexane phase was discarded). The washed methanolic extract was filtered using Whatman nº 4 filter paper and an aliquot of 100 µL of sample extract, BF or standard was mixed with 3 mL of 2% (*w*/*v*) sodium carbonate aqueous solution and 150 µL of 50% (*v*/*v*) Folin-Ciocalteu reagent. The mixture was incubated at room temperature in darkness for 1 h, and the absorbance at 765 nm was measured on a spectrophotometer (Perkin Elmer lambda 2 UV-VIS, Überlingen, Germany). Quantification was achieved using a gallic acid external standard calibration curve in the range of 0–1000 mg/L. The results were expressed as gallic acid equivalent (GAE)/100 g DW.

### 2.5. Determination of Antioxidant Capacity

Lyophilized microgreens were previously subjected to the same methanolic extraction process described above for total soluble polyphenols and BF were directly used.

#### 2.5.1. Trolox Equivalent Antioxidant Capacity Assay (TEAC)

TEAC assay measures the reduction of the radical cation ABTS by antioxidant compounds, and the spectrophotometric method proposed by Cilla et al. [36] was used. The ABTS^+^ radical cation stock solution was generated by chemical reaction with 7 mM ABTS and 140 mM K_2_S_2_O_8_ overnight in darkness at room temperature. Next, it was diluted in ethanol until an absorbance of 0.700 ± 0.020 at 734 nm and 30 °C to obtain the ABTS^+^ working solution. The optimal dilution of the samples to obtain a percentage of absorbance inhibition of approximately 50% was 1/3 (*v*/*v*) in ethanol. At the same time, Trolox standard solutions were prepared in a range of 0 to 300 µM. The absorbance of 2 mL of ABTS^+^ working solution was considered the initial point of reaction (A_0_). Then, diluted samples or Trolox standards (100 µL) were added immediately and the absorbance were measured after 3 min (A_f_). All readings were carried out in a thermostatized UV–vis spectrophotometer. The percentages of absorbance inhibition were obtained from the following Equation (4):

1 − (A_f_/A_0_) × 100 (4)

and were compared to Trolox standard curve to express the results as µM Trolox equivalents/100 g DW.

#### 2.5.2. Oxygen Radical Absorbance Capacity Assay (ORAC)

The ORAC assay measures the capacity of the antioxidant compounds to scavenge peroxyl radicals; the fluorimetric method described by Cilla et al. [36] was used. The reaction was carried out in a Multilabel Plate Counter VICTOR^3^ 1420 (PerkinElmer, Turku, Finland) with fluorescence filters for an excitation wavelength of 485 nm and an emission wavelength of 535 nm at 37 °C. The optimization of the assay parameters was required. Sodium fluorescein and freshly prepared AAPH solution were used at a final concentration of 0.015 and 120 mg/mL respectively. Samples were diluted 1/250 (*v*/*v*) and 20 µM Trolox was used as antioxidant standard. All of them were prepared with phosphate buffer (75 mM, pH 7.4). The final reaction consisted of 80 μL of fluorescein, 40 μL of AAPH and 80 μL of diluted sample, Trolox standard or phosphate buffer (blank) and the fluorescence was recorded every 5 min over 70 min (until the fluorescence in the assay was less than 5% of the initial value). The results were calculated considering the differences of areas under the fluorescence decay curve (AUC) between the blank and the sample over time, and were expressed as µM Trolox Equivalents/100 g DW.

### 2.6. Analysis of Minerals

The main macroelements (K, Ca, Mg) and oligoelements (Fe, Zn) were evaluated according to Cilla et al. [37,38]. Briefly, 1 g of each lyophilized microgreen was ashed in a muffle furnace (Heraeus, Eurotherm, Germany) at 450 °C for 48 h (the temperature being slowly increased at a rate of 50 °C/h). In the case of BF, 10 g were heated until complete evaporation before being introduced into the furnace. Next, 1 mL of concentrated nitric acid was added to the white ashes and heated on a hot plate to dryness. Immediately after, samples were dissolved in 3 mL of concentrated HCl and allowed to flux for 3.5 h. Then, the digest was filtered through Whatman nº 4 filter paper and the filtrate was diluted with ultrapure water to a final volume dependent on the total concentration estimated for each element, in both lyophilized microgreens and BF samples. Titrisol standard solutions of K, Ca, Mg, Fe and Zn were prepared in ultrapure water containing the same % of HCl used to dissolve ashes. Lanthanum oxide (La_2_O_3_) and cesium chloride (CsCl) at 0.1% (*p*/*v*) were added to samples and standards to eliminate possible chemical interferences of phosphate on calcium and to avoid potassium ionization, respectively.

Mineral concentrations were determined by flame atomic spectrometry (Thermo Scientific ICE 3000, UK) and the quantification of minerals was calculated from their standard calibration curves (mg/L): K (0.25–2.5), Ca (0.125–5.0), Mg (0.125–1.0), Fe (0.0625–5.0) and Zn (0.0625–2.5). The results were expressed as mg of each element/100 g FW. In addition, a dried hay powder (Certifed Reference Material BCR-129) was used to confirm the accuracy of the method. It was prepared and analyzed using the same procedure as that followed for the microgreen samples. The certified and experimental values were (mg/L) 640 ± 10 and 609 ± 4 for calcium, 145 ± 4 and 115 ± 1 for magnesium, 3380 ± 80 and 2850 ± 18 for potassium, 11.4 ± 0.0 and 13.4 ± 0.1 for iron and 3.2 ± 0.17 and 4.01 ± 0.10 for zinc, respectively. The coefficient of variation with regard to the precision for all minerals was in the range of 0.62–2.42%.

### 2.7. Statistical Analysis

All analyses were carried out in triplicate in at least two independent experiments, and data were expressed as mean ± standard deviation. Experimental data were subjected to one-way analysis of variance (ANOVA) to determine significant differences among samples composition. Tukey’s multiple range test, at a significance level of *p* < 0.05, was used. All analyses were performed with the software Statgraphics Plus 5.1 (Statpoint Technologies Inc., Warrenton, VA, USA).

## 3. Results and Discussion

### 3.1. Content and Bioaccessibility of Antioxidant Bioactive Compounds in Microgreens

The results of antioxidant bioactive compounds content in fresh microgreens and their bioaccessible fraction, as well as the bioaccessibility, are shown in Table 1. The concentration range of ascorbic acid in fresh microgreens was from 31 to 56 mg/100 g FW, which would provide between 38 and 70% of the recommended daily intake for vitamin C, justifying the inclusion of the nutritional claim “high vitamin C content” according to the Regulation (EU) 1924/2006, Annex II [39]. Kale microgreen contained the highest concentration, followed by broccoli, radish and mustard. The results of ascorbic acid content are within the ranges described in recent published data for microgreens of kale (28–66 mg/100 g FW), mustard (19–44 mg/100 g FW) and radish (25–68 mg/100 g FW) and lower than those found in broccoli (89 mg/100 g FW) [6,20,24]. Considering the National Nutrient Database for Standard Reference (USDA, 2018) [40] and data in the literature for adult plants (see Table 2), the ascorbic acid concentration in microgreen samples was higher for radish and lower for kale and mustard, while broccoli was within the range described (Table 2). On the other hand, the results obtained in the BF were 0.6–1.2 mg AA/100 g FW. The lowest content of ascorbic acid in BF was observed in the broccoli microgreen, while there were no statistically significant differences in kale, mustard and radish. These very low values seem to indicate a high loss of ascorbic acid, possibly due to instability at intestinal pH and oxidation in presence of oxygen. Although there are no bioaccessibility (BA) data for microgreens in the literature, similar vitamin C losses, i.e., greater than 95%, have been reported in pomegranate juice and in broccoli inflorescences after in vitro gastrointestinal digestion [41,42].

Regarding to total carotenoids content, the concentration ranged from 162 to 224 mg β-carotene/100 g DW. Radish microgreen showed the lowest value before the digestion process, and no statistically significant differences (*p* > 0.05) were found between broccoli, kale and mustard. For radish, lower (46–66 mg/100 g DW) and similar contents (85–200 mg/100 g DW) have been described [6,8,20]. For broccoli microgreens, a lower concentration of total carotenoids (118–209 mg/100 g DW) was reported in several studies, regardless of the growing system applied: hydroponic [17] or peat substrate [6,7]. In the case of kale, the value obtained was higher than the range described by Xiao et al. [20] (141–197 mg/100 g). As for mustard microgreens, different amounts of total carotenoids content were found (27–270 mg/100 g DW) [6,8,20], which was in agreement with our data. The developmental stage at harvest, light intensity during the growth period, or genotypic differences between species were suggested as important factors for the final carotenoid content in microgreens [6]. Overall, microgreens have been considered as good sources of β-carotene [8]. In addition, the four *Brassicaceae* microgreens analyzed showed extremely high total carotenoid concentrations compared to their mature counterparts (Table 2), and also in accordance with the 260-fold more β-carotene determined in cabbage microgreen versus the adult plant [5]. Very low contents of carotenoids in BF were observed, and therefore, minor BA were obtained (<0.15%). The same results were reported by Courraud et al. [58] in fresh spinach using HPLC. However, some studies reported a BA from 1 to 20% in broccoli and kale vegetables analyzed by HPLC [47,59,60]. The reason for the low BA of carotenoids in microgreens could be due to differences in the digestion method conditions and to the chemical structure adopted by these compounds into the plant matrix, since it has been hypothesized that carotenoids in crystalloid form would not be transferred to the micellar aqueous phase as they do in cabbage (*Brassicaceae* family) [60].

The total isothiocyanate concentration in microgreens ranged from 608 to 810 mg sulphoraphane/100 g DW (Table 1). Mustard and radish showed higher values than broccoli and kale. There is no data in the literature about isothiocyanates present in microgreens. However, Hanlon and Burnes [25] reported a range from 970 to 3762 mg/100 g DW in 8 varieties of 7-day-old radish sprouts. Regarding values of isothiocyanates in adult plants, the literature is also limited and variable, from 2–4 times less content in radish taproots versus radish microgreens to a wide difference of concentrations in broccoli florets (Table 2). Both the content in the BF ranging from 205 to 513 mg/100 g DW and the BA (31–63%) were double in radish compared to the rest of the samples. In general, the results of BA of total isothiocyanates in microgreens were similar to those described in mature cruciferous vegetables such as radish and mustard (43–72%) using the same spectrophotometric methodology [53]. The reduction of the total content of isothiocyanates during the digestion process could be due to the chemical transformations caused by the action of gastric pH, obtaining new non-detectable compounds (phenethylamines) for the analytical conditions [53].

Total anthocyanin content in microgreen samples were from 1.4 to 36.4 mg cyanidin-3-glucoside/100 g DW, following this increasing order: kale, radish, broccoli and mustard (Table 1). The scarce data in the literature about anthocyanin content are quite variable, from very few to hundred μg per g FW, often depending on the colour [7]. In this sense, 30 different anthocyanins responsible for the coloration of five *Brassica* microgreens with red to purple seed-leaves have been identified [9]. Regarding mustard, very different results have been previously reported. Two varieties of 19-day-old red mustard leaves grown by natural irradiance presented concentrations of 30 and 67 mg/100 g DW [46]. In contrast, values of 760 mg/100 g DW [23] and 1480 mg/100 g DW [22] have been described in mustard microgreens grown before applying LEDs and short-term red lighting. The anthocyanin concentration in broccoli was equal to one variety (13 mg/100 g DW), but much lower than the other one (208 mg/100 g DW) analyzed by Paradiso et al. [7]. In addition, for 7-day-old radish microgreens, anthocyanin content varied from not detected to 29 mg/100 g DW [25]. Differences in the concentration of anthocyanins have also been observed in adult stage of radish and in two varieties of red mature mustard (Table 2). No data in the literature were found for broccoli and kale adult vegetables. For all the studied microgreens, no anthocyanins were detected in the corresponding BF (Table 1). In this context, Pérez-Vicente et al. [41] suggested that anthocyanins could be metabolized into colorless substances, oxidized, or degraded, giving rise to other chemical compounds which are not detectable by the spectrophotometric differential pH method. Likewise, a complete degradation or non-detection of anthocyanin pigments in some golden apple varieties after in vitro gastrointestinal digestion and applying the pH differential method have been described [61].

Total soluble polyphenol content in the microgreens varied from 1890 to 2416 mg GAE/100 g DW, with the highest value for kale and the lowest for mustard (Table 1). Two non-hydroponic varieties of broccoli microgreens showed 1092 and 1163 mg GAE/100 g DW [7], while mustard microgreens total polyphenols ranged from 536 to 2800 mg GAE/100 g DW [22,23,46]. A recent study of 13 microgreen species concluded that the polyphenol composition profiles were significantly different across species [6]. In general, the total soluble polyphenols determined in the microgreens of the present study were within the range of their corresponding adult stage (Table 2). The identification of 164 polyphenols in five *Brassica* microgreens revealed more complex profiles and a greater variability in the content of polyphenols in microgreens compared to mature plants [9,62]. As for the content of soluble polyphenols in the BF (821–1448 mg/100 g DW) the lowest amount was observed in mustard while there were no statistically significant differences between broccoli, kale and radish. The decrease in BA, showing values from 43% to 70%, could be due to the slightly alkaline conditions reached after intestinal phase, together with possible interactions with digestive enzymes. No data are available in literature about BA in microgreens; nevertheless, our results were comparable to those obtained by Puangkam et al. [53] using the Folin Ciocaltou method for conventional vegetables of the *Brassicaceae* family including radish and mustard. Lower values of BA were determined by HPLC in broccoli flavonoids (11%) and for total polyphenols in raw kale (15%) or in kale subjected to different culinary techniques (7%) [42,55,60]. The determination of total soluble polyphenols by the Folin-Ciocalteu assay may present some interferences and limitations, but it offers a rapid chemical index. In addition, spectrophotometric methods have been regarded as useful screening techniques for comparison among samples providing an idea of the antioxidant capacity in the matrix [63]. The measurement of the phenolic profile, as well as that of other antioxidant bioactive compounds found in these microgreens through chromatographic analysis, could be interesting for future research.

The results of total antioxidant capacity determined by TEAC and ORAC methods in microgreens, their bioaccessible fractions and the percentage retained in the BF are shown in Table 3. The antiradical activity of fresh microgreens ranged from 422 to 493 and from 7579 to 9783 µM Trolox Eq/100 g DW for TEAC and ORAC assays, respectively. In general, broccoli showed a slight lower antioxidant capacity compared to the rest of microgreens. In contrast, the results of antioxidant capacity determined by DPPH method in six genotypes of microgreens showed the highest activity levels for broccoli microgreens [7]. The comparison of antioxidant activity is limited due to the different existing methods. According to the ORAC Database [51], higher values (from 2 to 8-fold) were found for mature broccoli, kale and radish compared to the microgreen samples we analyzed, and no data was available for mustard (Table 2). The antioxidant capacity in the BF varied from 78 to 138 (TEAC) and from 3646 to 7453 (ORAC) µM Trolox Eq/100 g DW. For TEAC method the highest value in BF was observed in radish, and the highest percentage retained in the BF resulted in both radish and mustard, while for the ORAC method, mustard and kale showed higher antioxidant capacities than broccoli and radish in BF and the highest antioxidant percentages retained in the BF. Different results of antioxidant capacity were observed in cruciferous vegetables (radish and mustard) subjected to a simulated gastrointestinal digestion using DPPH and FRAP methods with percentage retained in the BF of 59–69% and 12–28%, respectively [53]. These differences could be related to the compounds formed after digestion process, which are susceptible to various reactions with substrates and free radicals according to each antioxidant method, depending on the matrix. The decrease in the antioxidant capacity observed with both methods after gastrointestinal digestion, is attributable to the reduction in bioactive antioxidant compounds (ascorbic acid, total soluble polyphenols, total anthocyanines, total carotenoids and total isothiocyanates) previously discussed (Table 1). The decrease was more pronounced in the case of TEAC method showing percentage of antioxidant capacity retained in the BF between 19–28% values versus 48–82% observed with ORAC method.

### 3.2. Content and Bioaccessibility of Mineral Elements in Microgreens

The total content of mineral elements in the microgreens before and after gastrointestinal digestion and their BA are reported in Table 4. For all fresh microgreens (mg/100 g FW), the most abundant element was K (86–102), followed by Ca (31–40), Mg (11–13), Fe (0.30–0.39) and Zn (0.15–0.16). In general, the same order was observed in different studies about macro- and micro- mineral content for the same microgreen species here evaluated [6,7,10,19,21]. Among the 30 varieties of *Brassicaceae* microgreens grown in peat moss substrate evaluated by Xiao et al. [19] and expressed in mg/100 g FW, the range of K (176–365), Ca (41–88), Mg (28–60), Fe (0.47–0.72) and Zn (0.29–0.43) content in broccoli, kale, mustard and radish was higher than those found in this study. Similarly, also for broccoli microgreens grown on a mixture of peat [7] and compost [10] macro- and oligoelements were also higher (mg/100 g FW) (K: 249–422, Ca: 59–202, Mg: 21–40, Fe: 0.59–1.2, Zn: 0.30–0.73). However, when broccoli microgreens were obtained through two different hydroponic growing systems, similar results (mg/100 g FW) were found in K (79–101), lower in Ca (29–32) and higher in the rest of the elements analyzed (Mg: 33–36, Fe: 0.48–0.61, Zn: 0.47–0.53) [9]. As for kale, three cultivars grown in soilless media and harvested at five different development stages generally showed lower K, Ca, Mg, Fe and Zn content at the microgreen stage than at the baby leaf one, and fresh microgreens also showed lower concentrations of Ca and Mg than adults [21]. In general, the concentration of all macro- and oligo-elements measured in microgreen samples were lower than those found in mature plants (Table 2). In particular, K content was more than 30% lower than the average K content found in the adult counterparts. Furthermore, Renna et al. [13] demonstrated that in hydroponically grown microgreens K can easily be modulated by controlling the element concentration in the nutrient solution. Thus, microgreens produced with these specific conditions could be labeled with the nutritional claim of “reduced potassium” (Regulation 1924/2006) [39], and could be recommended for patients with impaired kidney function [13]. 

The highest BA for the three macroelements analyzed was found in mustard microgreens. In contrast, broccoli microgreens showed lower BA values for Ca and Mg. Although Fe and Zn could not be detected in the BF, a decrease in the amount of macroelements occurred after digestion process, high BA (34–61% for Ca, 59–73% for Mg and 80–90% for K) was observed. This fact could be probably ascribed to the low content of ascorbic acid and high content in total soluble polyphenols in the BF (substances that promote and inhibit BA of minerals, respectively) of broccoli, in contrast to mustard. There are no data in the literature about BA of mineral elements in microgreens. However, the values of Ca BA in conventional vegetables of *Brassicaceae* have been described in two different studies. Lucarini et al. [64] obtained 27–40% BA in cooked broccoli and kale and Kamchan et al. [65] showed 33–39% BA in two kinds of kale. These values are slightly lower than those in our study.

## 4. Conclusions

In general, the four hydroponic *Brassicaceae* microgreens produced in this study could be considered as good sources of minerals and antioxidant phytochemicals in a balanced human diet. In particular, they contain relevant amounts of vitamin C, higher levels of total carotenoids than adult plants, mineral and antioxidant bioactive compound contents comparable to other hydroponic microgreens, and low K, making them suitable for patients with impaired kidney disease.

In this study, bioaccessibility data for antioxidant bioactive compounds, total antioxidant capacity, and mineral elements in microgreens are provided for the first time. Radish and mustard showed the highest BF and BA values for antioxidant parameters, while broccoli and mustard provided the lowest and highest values for minerals, respectively. Despite the expected decrease in different compounds after the in vitro digestion process, the bioaccessible fractions of microgreens still contained remarkable total antioxidant capacities and bioactive compounds with potential beneficial local effects in the gastrointestinal tract. For future studies, determining the bioaccessibility of the antioxidant phytochemicals in more microgreen species, as well as their potential bioactivity in pre-clinical and human intervention studies, ought to be addressed.

## Figures and Tables

**Table 1:** Antioxidant bioactive compounds content in broccoli, kale, mustard and radish microgreens before and after simulated gastrointestinal digestion.

Microgreen	Total Content mg/100 g	Bioaccessible Fraction mg/100 g	Bioaccessibility (%)
*Ascorbic Acid* ^1^			
Broccoli	50.99 ± 1.91 ^b^	0.56 ± 0.09 ^b^	1.10 ± 0.17 ^d^
Kale	56.14 ± 1.04 ^a^	1.05 ± 0.09 ^a^	1.87 ± 0.17 ^c^
Mustard	30.67 ± 1.02 ^d^	1.14 ± 0.10 ^a^	3.73 ± 0.32 ^a^
Radish	45.43 ± 1.15 ^c^	1.19 ± 0.09 ^a^	2.61 ± 0.21 ^b^
*Total carotenoids (β-carotene)* ^2^			
Broccoli	221.80 ± 13.36 ^a^	0.18 ± 0.02 ^b^	0.08 ± 0.01 ^c^
Kale	217.54 ± 18.74 ^a^	0.12 ± 0.02 ^c^	0.06 ± 0.01 ^d^
Mustard	224.27 ± 9.35 ^a^	0.25 ± 0.02 ^a^	0.11 ± 0.01 ^b^
Radish	162.29 ± 5.50 ^b^	0.23 ± 0.03 ^a^	0.14 ± 0.02 ^ab^
*Total isothiocyanates (sulphoraphane)* ^2^			
Broccoli	633.11 ± 10.69 ^b^	204.51 ± 47.94 ^b^	32.30 ± 7.57 ^b^
Kale	608.23 ± 35.63 ^b^	207.18 ± 10.33 ^b^	34.06 ± 1.70 ^b^
Mustard	801.07 ± 51.16 ^a^	248.90 ± 25.75 ^b^	31.07 ± 3.21 ^b^
Radish	809.62 ± 27.83 ^a^	512.99 ± 33.97 ^a^	63.36 ± 4.20 ^a^
*Total anthocyanins (cyanidin-3-glucose)* ^2^			
Broccoli	12.66 ± 1.53 ^b^	ND	-
Kale	1.39 ± 0.43 ^d^	ND	-
Mustard	36.40 ± 0.46 ^a^	ND	-
Radish	5.57 ± 0.86 ^c^	ND	-
*Total soluble polyphenols (GAE)* ^2^			
Broccoli	2037.38 ± 103.10 ^b^	1427.98 ± 175.00 ^a^	70.09 ± 8.59 ^a^
Kale	2415.95 ± 109.34 ^a^	1447.72 ± 140.10 ^a^	59.92 ± 5.80 ^a^
Mustard	1889.76 ± 64.81 ^bc^	820.57 ± 31.00 ^b^	43.42 ± 1.64 ^b^
Radish	2111.19 ± 132.79 ^b^	1434.82 ± 62.34 ^a^	67.96 ± 2.95 ^a^

^1^ Data presented in fresh weight (FW). ^2^ Data presented in dry weight (DW). Data are expressed as the mean ± SD (*n* = 3). Different lowercase letters in the same column for each bioactive compound indicate significant differences (*p* < 0.05). ND: not detected. GAE: Gallic acid equivalents.

**Table 2:** Antioxidant bioactive compounds and mineral elements content in mature counterparts of the microgreens evaluated in this study.

	Broccoli	Kale	Mustard	Radish	References
*Antioxidant bioactive compounds*					
Ascorbic acid (mg/100 g FW)	13–110	70–93	70	15–39	[33,43,44,45]
Total carotenoids (mg β-carotene/100 g DW)	2–28	27	0.17–0.21	43	[45,46,47,48]
Total isothiocyanates (mg sulphoraphane/100 g DW)	5–2307	NA	NA	189–368	[25,33,49]
Total anthocyanins (mg cyaniding-3-glucoside/100 g DW)	NA	NA	34–67	ND-189	[25,46,50]
Total soluble polyphenols (mg GAE/100 g DW) *	167–3606	967–3010	300–1702	0.2–13,890	[25,33,43,44,45,46,51,52,53,54,55]
*Total antioxidant capacity (µM Trolox Eq/100 g DW)*					
ORAC *	4785–15,887	28,698–36,030	NA	15,021–76,638	[51]
TEAC *	26,200	36,200	NA	NA	[54]
*Mineral content (mg/100 g FW)*					
K	310–599	165–348	384	233–495	[21,40,43,56,57]
Ca	27–88	169–254	115	25–752	[21,40,43,56,57]
Mg	17–40	33–98	32	10–57	[21,40,43,56,57]
Fe	0.34–0.73	0.34–1.6	1.64	0.34–3.8	[21,40,43,56]
Zn	0.41–0.85	0.39–0.61	0.25	0.28–0.39	[21,40,43,56]

ND: Not detected. NA: Not available. FW: fresh weight. DW: dry weight. GAE: Gallic acid equivalents. * calculated in dry base from values of water content from the specifics references or from USDA and ORAC Database (2018) when necessary.

**Table 3:** Total antioxidant capacity before and after simulated gastrointestinal digestion in microgreens.

Microgreen	Total Content µM Trolox Eq/100 g	Bioaccessible Fraction µM Trolox Eq/100 g	Antioxidant Capacity Retained in BF (%)
*TEAC* ^1^			
Broccoli	421.81 ± 19.35 ^b^	78.39 ± 9.05 ^c^	18.58 ± 2.15 ^b^
Kale	493.21 ± 25.10 ^a^	98.69 ± 11.26 ^b^	20.01 ± 2.28 ^b^
Mustard	447.98 ± 11.55 ^b^	110.81 ± 18.57 ^b^	24.73 ± 4.15 ^a^
Radish	488.65 ± 19.20 ^a^	137.70 ± 11.30 ^a^	28.18 ± 2.31 ^a^
*ORAC* ^1^			
Broccoli	7578.89 ± 815.87 ^c^	3645.50 ± 281.21 ^b^	48.10 ± 3.71 ^c^
Kale	9782.57 ± 822.34 ^a^	7391.52 ± 1162.12 ^a^	75.56 ± 11.88 ^a^
Mustard	9090.15 ± 907.25 ^ab^	7452.51 ± 701.65 ^a^	81.98 ± 7.72 ^a^
Radish	9690.38 ± 935.81 ^a^	5258.94 ± 721.69 ^b^	54.27 ± 7.45 ^b^

^1^ Data presented in dry weight (DW). BF: bioaccesible fraction. Data are expressed as the mean ± SD (*n* = 3). Different lowercase letters in the same column in each antioxidant capacity assay indicate significant differences (*p* < 0.05).

**Table 4:** Mineral content before and after simulated gastrointestinal digestion in microgreens.

Microgreen	Total Content mg/100 g	Bioaccessible Fraction mg/100 g	Bioaccessibility (%)
*Potassium* ^1^			
Broccoli	86.21 ± 3.23 ^d^	71.81 ± 2.63 ^b^	83.30 ± 3.06 ^ab^
Kale	100.97 ± 2.02 ^b^	88.96 ± 2.30 ^a^	88.30 ± 2.28 ^ab^
Mustard	101.71 ± 1.10 ^ab^	91.82 ± 2.07 ^a^	90.27 ± 9.26 ^a^
Radish	95.04 ± 4.65 ^c^	76.15 ± 0.12 ^b^	80.13 ± 0.11 ^b^
*Calcium* ^1^			
Broccoli	37.38 ± 2.07 ^b^	12.67 ± 0.12 ^c^	33.91 ± 0.15 ^b^
Kale	40.38 ± 0.60 ^ab^	22.48 ± 0.18 ^a^	55.67 ± 0.17 ^a^
Mustard	32.20 ± 2.09 ^c^	19.8 ± 5.78 ^ab^	61.48 ± 17.94 ^a^
Radish	31.02 ± 1.07 ^c^	14.84 ± 0.15 ^bc^	47.85 ± 0.18 ^ab^
*Magnesium* ^1^			
Broccoli	11.95 ± 0.35 ^b^	7.03 ± 0.56 ^c^	58.83 ± 4.66 ^b^
Kale	11.21 ± 0.15 ^c^	7.87 ± 0.26b ^c^	70.26 ± 2.30 ^a^
Mustard	12.87 ± 0.19 ^a^	9.36 ± 0.69 ^a^	73.41 ± 6.36 ^a^
Radish	11.21 ± 0.17 ^c^	8.12 ± 0.42 ^b^	72.42 ± 3.79 ^ab^
*Iron* ^1^			
Broccoli	0.39 ± 0.03 ^a^	ND	-
Kale	0.39 ± 0.01 ^a^	ND	-
Mustard	0.32 ± 0.02 ^bc^	ND	-
Radish	0.30 ± 0.02 ^c^	ND	-
*Zinc* ^1^			
Broccoli	0.15 ± 0.04 ^a^	ND	-
Kale	0.16 ± 0.04 ^a^	ND	-
Mustard	0.15 ± 0.03 ^a^	ND	-
Radish	0.15 ± 0.02 ^a^	ND	-

^1^ Data presented in fresh weight (FW). Data are expressed as the mean ± SD (*n* = 3). Different lowercase letters in the same column for each mineral compound indicate significant differences (*p* < 0.05). ND: not detected.
